# Mr. Ring, on Inoculation

**Published:** 1805-08-01

**Authors:** John Ring

**Affiliations:** New Street, Hanover Square


*New Street, Hanover Square,*

*July* 16, 1805.

I am, &c.
JOHN RING.

---

### *To the Editors of the Medical and Physical Journal.*

Gentlemen,

In the year 1801, Dr. Chrestien, of Montpellier, published a work, entitled, "A Treatise on the Inoculation of the Small-pox, with some reflections on that of the Cow-pox," in which are the following remarks:

" Dr. Tandon, a physician of long experience, informs me, that in the year 1749, at least six thousand out of thirty-two thousand persons, of which the population of this city consists, died of the small-pox.

" The claveau sur les moutons, which may be called the sheep-pox, resembles the small-pox in its symptoms, its progress, and its mortality. I have frequently inoculated sheep with it, and with success.

" I have only seen a co-existence of the small-pox and the measles three times. Authors furnish us with a number of examples. The most remarkable are those related by Bergius; who says, 'Seven children were inoculated at Stockholm at the same time, and in the same house; when the measles prevailed in that city, and attacked them all nearly at the same period. The eruption of the measles took place in one before, in others at the same time, and in others after that of the small-pox, without any unfavourable event.' In these cases, when the measles appeared first, they suspended the small-pox; but when the small-pox appeared first, it did not suspend the measles."

Dr. Chrestien inoculated himself in the arm with ovine matter; and excited a pustule, with pain in the throat and chest; acute, but of short duration. The exsiccation of the pustule was complete, within three days from the time of inserting the matter. He says, "No prudent man will repeat repeat this experiment, the sheep-pox being frequently mortal, and producing its effects particularly in the windpipe and the chest, where they occasion a considerable suppuration; as I have learned by opening a number of sheep, which have died of this disease.

"Many years ago I inoculated two sheep with variolous matter, and an eruption was produced; nevertheless, when exposed to the infected flocks, they caught the distemper."

When Dr. Chrestien published his Treatise, he was not perfectly convinced of the full advantages of vaccination; but his doubts are stated with great moderation and candour.

In your Journal for September, 1802, is an account of some experiments made for the purpose of proving, that the small-pox is a disease common both to men and brutes, by Professor Viborg, of the Veterinary College at Copenhagen. It there appears, that the Professor produced a local infection in lambs, but could not produce a general eruption. In sheep he could not produce even a local infection, notwithstanding he had attempted it by repeated experiment. He nevertheless was of opinion, that it was possible to be done. The experiments of Dr. Chrestien prove this opinion to have been well founded.

Prof. Viborg observes, that it is interesting to examine the relation between the sheep-pox and the small-pox; to ascertain, whether the small-pox can be prevented by the inoculation of the sheep-pox; and whether the sheep-pox can be communicated to the human species, and to other animals. This question also is now decided, as far as relates to the human species; and in a way that must deter any rational man from trying a similar experiment.

---

Two other cases of the small-pox have occurred since my last communication, in patients vaccinated by me. One of them is a child of Mr. Pearse in Great York Mews, Baker Street, who was inoculated four years ago, with matter taken on the tenth day. I have long avoided to take matter at so late a period; but at the same time must acknowledge, that in this case I frequently saw the pustule, which appeared perfect, and to go through its regular course.

The subject of the other case is a child of Mr. Stokes, No. 12, Orange-court, Swallow-street, whom I inoculated five years ago, at a time when it was not thought of much consequence at what period matter was taken, provided a pustule, resembling a vaccine pustule, took place. Such a pustule a pustule took place in the present instance, and afforded matter for a considerable number of other successful inoculations; and those who were thus inoculated, have resisted the infection of the small-pox. I cannot, therefore, but consider it as having been a genuine case. The small-pox, in this instance and the former, as well as others, is so very benign for the natural sort, that I conclude it is modified and mitigated, though not totally prevented, by the preceding vaccination.

In the last Number of the Medical and Chirurgical Review is the case of a daughter of Mr. Scott, No. 28, London-street, Fitzroy-square, who is there said to have been brought to the Small-pox Hospital on the 5th of May, with an eruption which had every appearance of the small-pox. It is also stated, that she sickened on the Tuesday preceding, and continued ill with fever until Thursday, when she had a slight fit; that on the following morning the eruptions appeared; and that the disease was caught from a younger child, then recovering from the small-pox, who had been inoculated at the Small-pox Hospital.

This statement is partial, and liable to mislead. It is true, the younger child was inoculated at the Small-pox Hospital; it had the disease in a very severe manner; and I expressed my surprise at hearing that the mother frequently carried such a dreadful object through the streets to the Small-pox Hospital: on which she informed me, that she carried it *under her apron.* Hence it appears, that these masses of contagion, which have so justly been compared to foxes with firebrands at their tails, are carried through the streets of the metropolis more frequently than the public suspect; and the danger is aggravated by concealment. It is therefore imprudent to let other children be carried abroad in this town or its vicinity, till after inoculation.

I shall now add some further particulars, respecting the case of the child who had been vaccinated, and in whom the varioliform eruption appeared. These particulars were communicated by the father and mother of the child.

The small-pox on the younger child had been very thick and confluent, and great numbers of the pustules broken at an early period, while the fluid they contained was watery. The other child played with him, and for many nights lay with him, while he was in this state; of course, she must have had active variolous matter applied to her in a variety of modes: First, directly from the infant; secondly, condly, from the sheets in which they lay; and, thirdly, from the hands of the mother, who nursed them both.

The following is a memorandum of the particulars of the case, made on the afternoon of the same day on which she was carried to the hospital; and taken from the account given to me by the parents.—On Tuesday, April 30th, Grace Scott appeared indisposed. *At the same time she complained very much of her eye; beneath which a scratch appeared. The eyelids swelled more and more for the space of two days; and she was in great pain.*

"On the Friday following, two eruptions appeared on the scratch; and another in the same direction; and at the same time smaller eruptions began to appear in different parts of the body."

These eruptions turned *on the fourth day.* They appeared to me to be no more than miliary eruptions, such as occurred in the case of Mr. Grant's child, related by Mr. Goldson, or in the case related by Dr. Rollo. They never became pustular; nor appeared to contain any fluid; of course, they were not small *pocks.* They will serve, however, as well as the best, for the Medical and Chirurgical Review; where it has too long been the custom to bring forward every thing which appears after vaccination, bearing the least resemblance to the small-pox, in order to prejudice the public.

As one proof of this, a case is published in the same number with the preceding, in which it is said, "the arm inflamed regularly, and the patient sickened on the 8th or 9th day; and continued to be very ill for two days. A copious *rash* came out; but went off in a couple of days. The arm is now scabbed; and there are marks of *two or three pimples* on the margin. The scab is *circular,* as *dark in colour as the vaccine;* but *neither so large, nor elevated above the surrounding skin. No pustules appeared on any part of the body.*"

It is well known, that the scab here described is common in those who are put to the test after vaccination. It is also well known, that when those who have had the small-pox or the cow-pock are put to the test of variolous inoculation, a local pustule is sometimes produced, attended with constitutional indisposition, and followed by a rash, or an eruption of pimples. But it is an abuse of language, an insult to common sense, and an injury to the cause of truth, to call such an eruption *pocks* or *pustules.*

As to the eruption in Mr. Scott's child, it was seen on the fourth day by Mr. Sarjeant, of Prince's Street, Cavendish Square; who declared, that had he been called in to the case without being informed of the small-pox having been in the house, he should not have considered it to be of the variolous kind.

Mrs. Scott herself, who was as much indisposed as her daughter, had fourteen *pustules*; several of which were large; but others were ruptured at an early period by nursing her children. She declares that the eruption in her daughter totally disappeared in six days.

It is rather surprising, after all that has been done, said, and written, that the public in general, and even some members of the medical profession, appear to be still ignorant of the local as well as constitutional effects, which are sometimes produced by the mere contact of variolous matter. In addition to the very considerable number of instances of this kind, which I have already published, I now beg leave to state the following.

Mrs. Young, No. 8, Exeter Place, Brompton, having a nurse-child, in whom, after vaccination, some pustules broke out round the mouth, in consequence of being suckled at the same breast with her own infant, when labouring under the small-pox; I remarked, that the same thing takes place, in some instances, from the application of variolous matter to the skin of those who have had the small-pox. To this she replied, that she knew it very well; for when she nursed her former child in the small-pox, her fingers were so affected by handling the matter, that all her nails came off.

Mrs. Cottis, of Great York Mews, Baker Street, from the same cause, had seven pustules on her face, and one or two on her arm, although she had had the small-pox before; and this second eruption was attended with considerable constitutional indisposition for many days. She had a violent pain in her head, back, and limbs; great fever, and intolerable thirst. Yet the eruption was evidently local.

Mrs. Read, of Fulham, who had also had the small-pox before, when nursing her child in that disorder, had nine large pustules; some of which were very painful. Her face swelled, and she was almost blind three days. She had also a violent sore throat.

Mrs. Watts, of Fulham, had the small-pox severely when four years old, and was in great danger of losing her life by the disorder; nevertheless, when lately nursing her child, she again had a considerable number of pustules, attended with great swelling and inflammation of the the face. She was extremely ill; and obliged to keep her bed two days, and to have medical attendance.

These cases, if there were no others on record, ought to inculcate a little caution, in judging of the occurrence of the small-pox after vaccination. But, in order to strengthen my argument, I shall add one more: the same which I alluded to in my letter to Mr. Grant of Portsmouth, as noticed by Mr. Goldson. It occurred in a child of Mr. Merriman, of Queen Street, Berkeley Square; who has favoured me with the following particulars.

The child was vaccinated in September, 1802, at the age of a month; and the vesicle went through all its stages in the most regular manner. There was but little constitutional indisposition; yet sufficient to satisfy Mr. Merriman, that she was secure from the future infection of the small-pox. Several children were inoculated from her, all of whom had the genuine cow-pock; and, after the scab fell off, a proper indentation was left on the arm.

In March, 1803, Mr. Merriman inoculated her with variolous matter, in order to put her to the test. On the second day a small eminence appeared, which increased till the ninth, when a severe shivering took place, followed by so much fever, as to cause an alarm. This, however, began to diminish on the next day.

At this period there was a considerable cluster of pustules round the inoculated part; and an eruption of about ten other pustules, if they deserved that name, in different parts of the body. These soon maturated, and most of them died away in three days; but one or two remained for five days.

Twelve or more medical friends, among whom were Dr. Jenner and Mr. Ring, saw the child under these circumstances; who agreed with Mr. Merriman in thinking, that the eruption was merely the effect of cutaneous irritation, and not the small-pox. Nevertheless, in order to decide this point, matter was taken from one of the pustules, and inserted into the arms of two children, who had never had either the small-pox or the cow-pock; but it produced no effect.

It was remarked by several medical gentlemen, particularly by Mr. Hemming, that the scab, formed in this case, was very different from that which takes place in the ordinary inoculation of the small-pox; and it is observable, that the indentation so nearly resembles that which is left by the inoculation of the cow-pock, as to be scarcely distinguishable from it. The arm continued sore for some weeks; with a free discharge of matter. The event of this case has ever since deterred Mr. Merriman from putting his vaccine patients to the test of variolous inoculation.

"Subsequent occurrences have proved, that this child's skin is peculiarly irritable. The ulcerations remaining after a blister, which a severe illness rendered necessary, could not be healed, by the greatest attention, for a considerable space of time. On this occasion also, there was an eruption of pimples, by no means confined to the vesicated part."

While I thank Mr. Merriman for this case, which he was so obliging as to communicate at my request, I beg leave also to acknowledge the valuable information I have received from perusing his remarks on that subject, just published; and his able vindication of the practice.

If we cannot prove vaccination to be perfect and infallible, let us at least recollect, that no other practice is perfect or infallible. If a few spots now and then appear on the bright orb of this new-discovered luminary, let us recollect, that the sun itself has a few spots.

——— nitent ubi plurima, non ego paucis

Offendar maculis.

I shall here add some other instances of failure in the inoculation of the small-pox; a practice which it has been so much the fashion of late to represent as infallible.

A child of Mr. Hookham, in Old Bond-street, was inoculated for the small-pox, and supposed to be safe from future infection; but afterwards had the disorder in the natural way.

A child of Mr. Butts, a broker in Oxford-street, near Blenheim-steps, was inoculated for the small-pox when three months old. The arm rose, and she had several convulsive fits previous to the eruption, which left pits behind. Nine months after, she caught the small-pox; and had it so severely, that it was with difficulty she recovered. No one who sees the marks remaining from this last attack, can doubt that the disease really was the small-pox.

Mrs. Fennel, of Fulham, mother of Mrs. Watts, whose case of local small-pox, in an uncommon degree, is before related, informed me of an instance of a young woman, who, on coming to London, many years ago, was inoculated at the Small-Pox Hospital, and supposed to have the disorder in a regular way. She afterwards lived in service at Mr. Twigg's, in Gutter Lane, where she caught the small-pox, and died of it.

Mrs.

Mrs. Linforth, at No. 63, King-street, Golden-square, informed me of a young woman, who had been inoculated in the country, and lived with a grocer in Swallow-street, to whom the same melancholy event occurred.

But I have now before me a letter from the Rev. Mr. Jenner, written on the 4th of last June, in which he mentions a more recent case. This happened about the same time when a similar case occurred in the family of the Earl of Westmeath.

" Miss Price, niece of Mr. Whitebrook, wine cooper, in Greek Street, Soho, was inoculated for the small-pox, when an infant, by the late Dr. Barwis, of Devizes, at the time of a general inoculation. She had the disorder in a satisfactory manner; and some marks of it are still visible in her face.

" About the latter end of April she sickened; and had a pretty full eruption of the natural small-pox; of which she is just recovered. Her mother, from the recollection of her daughter's having the small-pox by inoculation, could not be convinced that this last eruption was the small-pox, till its complete character put the matter beyond dispute.

" Upon inquiry, I found she had the chicken-pox when a child; and, without this evidence, it is not probable Dr. Barwis could have been mistaken in the matter he took, when the small-pox prevailed in an extensive district. The period between the first and second cases of small-pox was eighteen years.

" In this instance, the disease was caught by receiving money from the hand of a child labouring under the small-pox,—*probably one of Mr. Travers's Firebrands.*

Dr. Becu, professor of physiology at Wilna, who has just left this metropolis, after residing here several months, informed Dr. Jenner of two cases of the small-pox a second time. The patients were attended each time by the same physician, who was a man of the first eminence.

Dr. Becu also informed me, that the small-pox has proved extremely fatal in his father's family. Eight of his brothers and sisters fell victims to that dreadful distemper; and he himself narrowly escaped. One brother only remained, whom Dr. Becu has rescued from the ravages of that devouring pestilence by vaccination. Such was the auspicious dawn of the practice in Lithuania; where this learned and enlightened practitioner has vaccinated almost two thousand persons with his own hands.

I am, &c.

